# Role of Transcerebellar Diameter and Transcerebellar Diameter/Abdominal Circumference Ratio in Assessing Fetal Growth and Diagnosing Intrauterine Growth Restriction

**DOI:** 10.7759/cureus.62713

**Published:** 2024-06-19

**Authors:** Challa Anil Kumar, Satyanarayana Kummari, Bagadi Lava Kumar, Krushi Mogadali

**Affiliations:** 1 Department of Radiology, Great Eastern Medical School & Hospital, Srikakulam, IND; 2 Department of Radiology, All India Institute of Medical Sciences, Nagpur, IND; 3 Department of Obstetrics and Gynaecology, Bangalore Baptist Hospital, Bangalore, IND

**Keywords:** fetal growth restriction, intrauterine growth retardation, small for gestational age (sga), abdominal circumference, appropriate for gestational age (aga), intrauterine growth restriction (iugr), transcerebellar diameter, tcd/ac ratio, tcd

## Abstract

Background

In obstetrics, accurately determining gestational age (GA) is a critical aspect of managing pregnancy and evaluating fetal growth and development. Intrauterine growth restriction (IUGR) is characterized by the failure of the fetus to reach its potential growth. Early detection of IUGR is crucial for optimal obstetric care to reduce fetal complications and neonatal morbidity and mortality. The purpose of the current research is to determine the role of transcerebellar diameter (TCD) and the TCD/abdominal circumference (AC) ratio in assessing fetal growth and diagnosing IUGR.

Methods

In the sample, there were 600 expectant mothers with GA exceeding 28 weeks. We measured TCD and AC and then calculated the TCD/AC ratio. We used IBM SPSS Statistics for Windows, V. 22.0 (IBM Corp., Armonk, NY), for statistical analysis. The data was subjected to statistical tests, including Pearson's correlation coefficient, coefficient of determination, and tests of validity.

Results

The current research demonstrates a strong linear correlation between TCD and GA. Additionally, there was no notable disparity in TCD measurements between normal and IUGR fetuses with the same GA. There was an insignificant relationship between the TCD/AC ratio and GA, with a constant TCD/AC ratio in the third trimester of pregnancy in normal fetuses. The mean TCD/AC ratio was 14.72±0.89 (mean±standard deviation), and a cut-off value of 16.5 was determined to diagnose IUGR.

Conclusion

TCD can serve as a reliable measure for GA estimation during the third trimester in pregnant women with uncertain last menstrual period (LMP) or no dating scan and IUGR fetuses. In diagnosing IUGR, the TCD/AC ratio has demonstrated greater sensitivity, specificity, positive predictive value (PPV), and negative predictive value (NPV). The TCD/AC ratio is a GA-independent measure that can be used to diagnose IUGR.

## Introduction

In obstetrics, accurately determining gestational age (GA) is a critical aspect of managing pregnancy and evaluating fetal development. Precision in this estimation is vital, as errors can lead to either preterm or postterm delivery. Moreover, in cases where the estimated date of delivery (EDD) is uncertain, there is a considerably higher likelihood of perinatal mortality [1-3]. Intrauterine growth restriction (IUGR) is characterized by a fetus that fails to reach its growth potential. In medical terminology, IUGR is typically defined as the estimated fetal weight (EFW) falling at or below the 10th percentile for GA at a specific moment during pregnancy, along with abnormal Doppler values. IUGR affects around 3-5% of pregnancies, although the exact percentage may vary depending on the specific group being studied. The prevalence of this condition in the general population varies from 3% to 10% [1-3]. After premature birth, IUGR is the second-most common cause of death among perinatal patients. Importantly, IUGR is responsible for 52% of stillbirths, and it can be directly linked to 10% of neonatal deaths [4,5]. Furthermore, a small for gestational age (SGA) status, which implies growth below the 10th percentile, is associated with up to 72% of fetal deaths that cannot be explained by any other factor [4]. In addition, the rates of neonatal morbidity and mortality among newborns who are impacted by IUGR are three to 20 times greater than the rates among neonates who do not experience IUGR [6].

The most frequently employed criteria for assessing fetal growth encompass measurements such as biparietal diameter (BPD), head circumference (HC), abdominal circumference (AC), and femur length (FL). Among these parameters, AC stands out as the most reliable predictor of IUGR. However, it is crucial to note that the correlation among these parameters is contingent upon precise knowledge of GA. The challenge of distinguishing between fetuses that are appropriately sized for their appropriate for gestational age (AGA) and those that are smaller for their GA becomes difficult when there is uncertainty regarding the GA of the fetus [3].

The transcerebellar diameter (TCD) represents the maximum transverse diameter of the fetal cerebellum. The fetal cerebellum can be observed through ultrasound (USG) as early as 10-11 weeks of pregnancy. It exhibits a linear correlation with GA from the second trimester onward, meaning it grows in proportion to the stage of pregnancy. Due to the fact that the fetal cerebellar hemispheres are located in the posterior cerebral fossa, which is an area that is resistant to external forces and growth variations, this measurement is extremely reliable for determining the GA of the fetus. Despite IUGR affecting all fetal biometric parameters, the cerebellum's size remains relatively unaffected, resulting in minimal changes in the TCD. Consequently, TCD serves as a more reliable parameter for determining GA [7]. Fetal AC is among the initial parameters that exhibit changes when fetal growth issues arise. Consequently, a TCD/AC ratio, unaffected by GA, emerges as a valuable tool for predicting IUGR [8].

The TCD/AC ratio is a reliable measure for determining fetal growth as it maintains consistency beyond 14 weeks of gestation. This makes it independent of GA and an excellent factor for assessing fetal growth [9]. Therefore, variations in the TCD/AC ratio can be used as a sensitive indication of IUGR. Disparities in TCD/AC ratios and their sensitivity in detecting IUGR across various studies may stem from differences in the populations under investigation. Given that biometric parameters can vary due to genetic and racial factors, it is essential to either create population-specific nomograms or prospectively validate an existing nomogram for accurate clinical utilization [10,11]. To our knowledge, there are only a few comprehensive studies determining the role of TCD and the TCD/AC ratio in assessing fetal growth and diagnosing IUGR in South India. The purpose of the current research is to determine the role of TCD and the TCD/AC ratio in assessing fetal growth and diagnosing IUGR.

## Materials and methods

Six hundred antenatal women at 28-40 weeks of GA, referred to the Obstetrics and Gynaecology (OB/GYN) Department of Bangalore Baptist Hospital, were included in the research. The research was a prospective observational, cross-sectional study. The research was conducted for two years. The research obtained approval from the Institutional Review Board and Ethics Committee (IRBEC) of Bangalore Baptist Hospital (approval number: IRBEC/BBH/108).

Inclusion criteria

Antenatal women with excellent dates, with GA exceeding 28 weeks, and carrying a live singleton pregnancy were added to the research.

Exclusion criteria

Antenatal women with anomalous babies, multiple gestations, unreliable dates, and deliveries outside of Bangalore Baptist Hospital and those patients who stated that they did not wish to take part in the research were excluded from the research.

Sample size

It was determined as a result of an anticipated prevalence of 15%, which led to a sample size of 600 participants at a confidence level of 95%.

Study procedure

**Figure 1:**
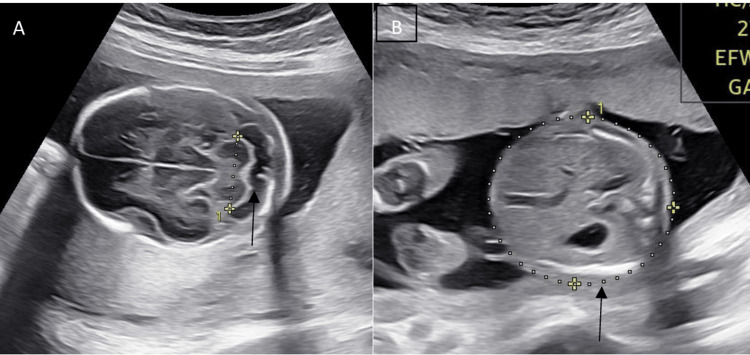
Measurement of ultrasound parameters Ultrasonographic measurement of (A) TCD (black arrow) and (B) AC (black arrow) TCD: transcerebellar diameter; AC: abdominal circumference

Data collection employed a questionnaire and USG scan reports. A single radiologist performed the measurements to decrease the inter-observer variability. To decrease the intra-observer variability, the average value of two measurements was used. Following informed consent from eligible participants, a semi-conservative proforma was completed, measuring TCD and AC and calculating the TCD/AC ratio, along with recording other scan parameters like BPD, HC, FL, and EFW. After delivery, information regarding the baby's birth weight, any complications during labor (e.g., meconium staining, fetal distress), and mode of delivery was documented (Figure 1).

Statistical analysis

The data that was collected was imported into Microsoft Excel 2019 and subjected to statistical tests including Pearson's correlation coefficient, coefficient of determination, and tests of validity such as sensitivity, specificity, positive predictive value (PPV), and negative predictive value (NPV). We used IBM SPSS Statistics for Windows, V. 22.0 (IBM Corp., Armonk, NY), to carry out the statistical analysis. We conducted a regression analysis to compare each USG-measured parameter, such as TCD, AC, and TCD/AC ratio, with the GA of the fetus. We also used correlation coefficients to compare each USG-measured parameter with the GA. Categorical variables were encoded with numerical values and percentages, whereas quantitative variables were displayed as the mean±standard deviation. The significance level was established at p=0.05, and any value equal to or less than 0.05 was considered statistically significant.

## Results

Six hundred and forty-three antenatal women with GA exceeding 28 weeks were added to the study. During follow-up, 43 antenatal women were lost, and they were not included in the study. The remaining 600 expectant mothers were included in the statistical analysis. The women's ages ranged from 20 to 40 years, with an average age of 27.42±3.71 years (Table 1).

**Table 1:** Age distribution in antenatal women

Age (in years)	Frequency	Percentage (%)
20-25	224	37.33
26-30	296	49.33
31-35	68	11.33
36-40	12	2.00
Total	600	100

**Figure 2:**
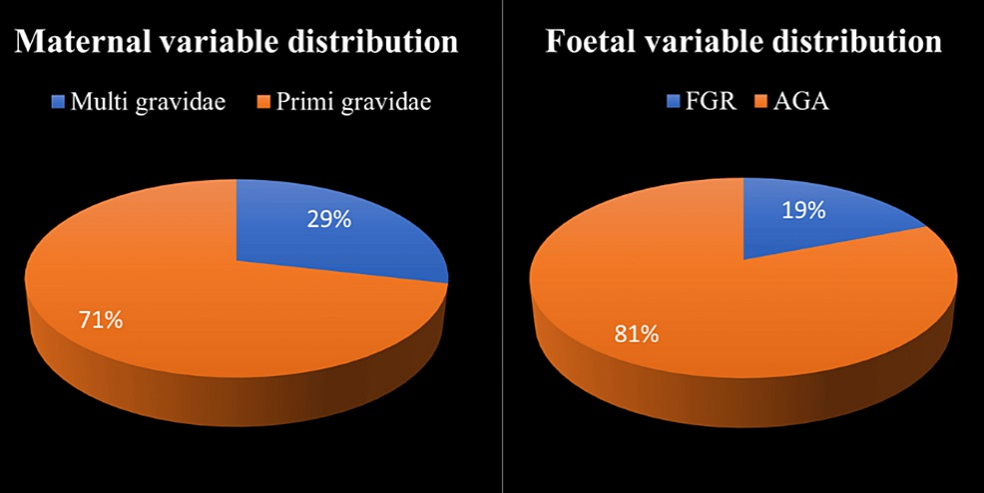
Maternal and fetal variable distribution: (A) parity of the antenatal women and (B) in utero growth of the fetuses FGR: fetal growth restriction, AGA: appropriate for gestational age

Out of 600 women, 428 (71.33%) were primigravidae. AGA was seen in 485 (80.83%) out of 600 antenatal women, and fetal growth restriction (FGR) was seen in 115 (19.16%) out of 600 antenatal women. The mean birth weight and GA of babies at delivery were 2.32 kg and 37.4 weeks, respectively (Figure 2).

The study shows a substantial linear relationship between the TCD and GA (r=0.9701; p=0.0001), a strong association between AC and GA (r=0.9583; p=0.0001), and a significant association between TCD and AC (r=0.9498; p=0.0001) (Table 2 and Table 3). The study demonstrated a poor correlation between the TCD/AC ratio and GA (r2=0.1635; p>0.05) with a constant TCD/AC ratio in the third trimester (Table 3).

**Table 2:** Correlation between different fetal variables GA: gestational age; TCD: transcerebellar diameter; AC: abdominal circumference

GA	Correlation coefficient (r)	P-value
Correlation between GA and TCD	0.9701	0.0001
Correlation between GA and AC	0.9583	0.0001
Correlation between TCD and AC	0.9498	0.0001

**Table 3:** Correlation between different fetal variables GA: gestational age; TCD: transcerebellar diameter; AC: abdominal circumference

GA	Coefficient of determination (r^2^)	P-value
Correlation between GA and TCD	0.9410	0.0001
Correlation between GA and AC	0.9183	0.0001
Correlation between TCD and AC	0.9021	0.0001
Correlation between GA and TCD/AC	0.1635	>0.05

In the current research, the TCD/AC ratio was less than 16 in most of the fetuses with normal growth and more than 16 in most of the fetuses with growth restriction. The mean TCD/AC ratio was 14.72±0.89 (mean±SD), and a cut-off value of 16.5 was determined to diagnose IUGR. With a cut-off value of 16.5, the TCD/AC ratio has demonstrated a sensitivity of 88.6%, a specificity of 94.5%, a PPV of 90.8%, and an NPV of 88.9% in detecting IUGR (Table 4).

**Table 4:** Diagnostic values of the current research PPV: positive predictive value; NPV: negative predictive value

Diagnostic values of the current research	
Sensitivity	88.6%
Specificity	94.5%
PPV	90.8%
NPV	88.9%

## Discussion

The term "intrauterine growth restriction" (IUGR) refers to a condition in which the EFW at a certain point in time during pregnancy is at or below the 10th percentile for the GA, along with abnormal Doppler values. In cases of IUGR caused by uteroplacental insufficiency or asphyxia, fetal blood flow becomes centralized, prioritizing the brain over other body parts [1,2]. BPD, HC, AC, and FL are the most commonly used parameters for assessing fetal growth (Figure 1). However, these parameters can only be correlated accurately when the GA is exactly determined [8,9]. AC is regarded as a critical measure for the early diagnosis of IUGR. However, to accurately predict IUGR, it is crucial to have precise information about the last menstrual period (LMP) or undergo a dating scan in the first trimester [1,3]. TCD measurements are reliable for determining true GA in such situations. The TCD/AC ratio is a reliable parameter for determining fetal growth as it maintains consistency beyond 14 weeks of gestation. This makes it independent of GA and an excellent factor for assessing fetal growth [9]. Consequently, a TCD/AC ratio, unaffected by GA, emerges as a valuable tool for predicting IUGR [9-11].

In the current research, most of the antenatal women, 296 (49.33%) out of 600, were aged between 26 and 30 years (Table 1); 428 (71.33%) out of 600 antenatal women were primigravidae; out of 600 antenatal women, 485 (80.83%) were AGA; and 115 (19.16%) were FGR (Figure 2). In the current research, the incidence of FGR was 19.16%, comparable to the reported incidence of 24% by Srividya et al. [12].

The current research demonstrates a significant linear association between TCD and GA (r=0.9701; p=0.0001), a strong association between AC and GA (r=0.9583; p=0.0001), and a significant association between TCD and AC (r=0.9498; p=0.0001) (Table 2). The research conducted by Meyer et al. [13] found a significant association between TCD and GA (r2=0.9464), AC and GA (r2=0.9685), and TCD and AC (r2=0.9561). These observations bear a resemblance to the current research.

The current research establishes a linear relationship between TCD and advancing GA in fetuses with appropriate growth for their GA as well as fetuses with growth restriction. Furthermore, there was no notable disparity in TCD measurements between normal fetuses and those with IUGR at the same GA. According to the findings of the current study, the TCD is only mildly affected, even in cases where there is severe growth restriction. A study carried out by Chavez et al. demonstrated identical results [10]. This could be due to the fact that TCD is minimally impacted by FGR, supporting the hypothesis that the brain-sparing effect facilitates human cerebellar growth and makes it resistant to chronic hypoxemia [10].

In their study of 192 women, Nikolov et al. discovered a significant association between GA and TCD (r=0.98), BPD (r=0.96), and FL (r=0.98). They suggest that TCD should be used as a standard method for determining the GA of fetuses [14].

The current research found that there was a poor correlation (r2=0.1635; p>0.05) between the TCD/AC ratio and GA in the third trimester (>28 weeks) (Table 3). Consequently, although the TCD/AC ratio increased slightly as GA increased, this rise was negligible, indicating that the ratio would remain constant regardless of GA. Comparable results were found in the studies that were carried out by Srividya et al. and Bhimarao et al. [12,15]. In the research carried out by Bhimarao et al., it was found that there was an insignificant association between TCD/AC and GA (r2=0.13, p>0.05) after 20 weeks of gestation [15].

In the current research, the TCD/AC ratio was less than 16 in most of the fetuses with normal growth and more than 16 in most of the fetuses with growth restriction. In the current research, the mean TCD/AC ratio was 14.72±0.89 (mean±SD). The studies that were carried out by Agrawal et al., Srividya et al., and Haller et al. obtained results that are comparable to those seen in the current study [1,12,16]. In a study carried out by Agrawal et al., it was discovered that in 15 neonates with IUGR, the mean TCD/AC ratio was 14.17±0.89 during the second trimester (14-28 weeks) and 15.61±1.18 during the third trimester (>28 weeks). In the fetuses with normal growth, the TCD/AC ratio was significantly lower, with values at early and late gestations of 13.50±0.97 and 13.80±0.97, respectively (p<0.05 for both) [1]. In another study carried out by Srividya et al., it was discovered that the TCD/AC ratio is typically below 15 in most fetuses that are born at the appropriate GA. The incidence of this was found to be 78.9%. Furthermore, the TCD/AC ratio was measured to be 16.43±1.61 at 20-22 weeks and 14.71±1.36 at 32-34 weeks [12]. In the research carried out by Haller et al., it was discovered that the TCD/AC ratio was typically more than 15.5 in most of the fetuses with IUGR. The incidence of this was found to be 80%. Moreover, the mean TCD/AC ratio was measured to be 14.40±1.20 (mean±SD) [16].

The current research has established a cut-off value of 16.5 (mean±SD) for the TCD/AC ratio to detect IUGR. It bears similarities to the work of Agrawal et al., wherein 16.79 was determined as the cut-off value to detect IUGR [1]. The cut-off value in a different study by Srividya et al. was 16.07, which is also similar to the current research [12].

In the current research, with a cut-off value of 16.5, the TCD/AC ratio has demonstrated a sensitivity of 88.6%, a specificity of 94.5%, a PPV of 90.8%, and an NPV of 88.9% in detecting IUGR (Table 4). The research carried out by Srividya et al., Meyer et al., Bhimarao et al., and Devi et al. demonstrated comparable findings [12,13,15,17] (Table 5).

**Table 5:** Comparison of the TCD/AC ratio of the current study with previous studies PPV: positive predictive value; NPV: negative predictive value; TCD: transcerebellar diameter; AC: abdominal circumference

	Sensitivity	Specificity	PPV	NPV
Srividya et al. [12]	83.0	96.1	85.7	93.7
Meyer et al. [13]	83.9	96.2	94.5	88.2
Bhimarao et al. [15]	88.0	93.5	77.1	96.3
Devi et al. [17]	89.5	83.0	98.0	62.4
Present study	88.6	94.5	90.8	88.9

There are some limitations inherent in the current research. A limited sample size of antenatal women from a single institution was studied. It was a short-duration study. The current research did not include antenatal women with anomalous babies, multiple gestations, or unreliable dates. However, the implementation of more comprehensive research endeavours with a larger cohort would yield a more precise depiction of the matter under investigation.

## Conclusions

The current research demonstrates a strong linear correlation between TCD and GA. Furthermore, there was no notable disparity in TCD measurements between normal fetuses and those with IUGR at the same GA. TCD can be utilized as an independent measure to determine GA in the third trimester (>28 weeks) of pregnancy, particularly in cases where the LMP is unknown or no dating scan has been performed and in fetuses with IUGR.

There was an insignificant correlation between TCD/AC ratio and GA in the third trimester (>28 weeks), and a consistent TCD/AC ratio and a cut-off value of 16.5 could be utilized as a growth measure to diagnose IUGR. In diagnosing IUGR, the TCD/AC ratio has demonstrated greater sensitivity, specificity, PPV, and NPV. The current research concludes that the TCD/AC ratio is a parameter that is independent of GA and can be utilized to diagnose IUGR, particularly in cases of unclear GA, with excellent diagnostic accuracy.
